# Investigation on Synthesis, Stability, and Thermal Conductivity Properties of Water-Based SnO_2_/Reduced Graphene Oxide Nanofluids

**DOI:** 10.3390/ma11010038

**Published:** 2017-12-27

**Authors:** Xiaofen Yu, Qibai Wu, Haiyan Zhang, Guoxun Zeng, Wenwu Li, Yannan Qian, Yang Li, Guoqiang Yang, Muyu Chen

**Affiliations:** 1 School of Materials and Energy, Guangdong University of Technology, Guangzhou 510006, China; yuxiaofensophia@126.com (X.Y.); zenggx@gdut.edu.cn (G.Z.); wenwuli@hust.edu.cn (W.L.); qianyannanhit@126.com (Y.Q.); 13128276397@163.com (Y.L.); yanggq525@163.com (G.Y.); 15626289608@163.com (M.C.); 2 Guangdong Provincial Key Laboratory of Functional Soft Condensed Matter, Guangzhou 510006, China

**Keywords:** SnO_2_/rGO nanocomposite, solvothermal, nanofluids, thermal conductivity, dispersion stability

## Abstract

With the rapid development of industry, heat removal and management is a major concern for any technology. Heat transfer plays a critically important role in many sectors of engineering; nowadays utilizing nanofluids is one of the relatively optimized techniques to enhance heat transfer. In the present work, a facile low-temperature solvothermal method was employed to fabricate the SnO_2_/reduced graphene oxide (rGO) nanocomposite. X-ray diffraction (XRD), thermogravimetric analysis (TGA), X-ray photoelectron spectroscope (XPS), Raman spectroscopy, and transmission electron microscopy (TEM) have been performed to characterize the SnO_2_/rGO nanocomposite. Numerous ultrasmall SnO_2_ nanoparticles with average diameters of 3–5 nm were anchored on the surface of rGO, which contain partial hydrophilic functional groups. Water-based SnO_2_/rGO nanofluids were prepared with various weight concentrations by using an ultrasonic probe without adding any surfactants. The zeta potential was measured to investigate the stability of the as-prepared nanofluid which exhibited great dispersion stability after quiescence for 60 days. A thermal properties analyzer was employed to measure thermal conductivity of water-based SnO_2_/rGO nanofluids, and the results showed that the enhancement of thermal conductivity could reach up to 31% at 60 °C under the mass fraction of 0.1 wt %, compared to deionized water.

## 1. Introduction

With the rapid development of industry, heat removal and management is a major concern for any technology. Heat transfer plays a critically important role in many sectors of engineering, which especially has been widely used in refrigerators, heat exchangers, automobiles, and electronic devices, etc. Nowadays, utilizing nanofluids is one of the relatively optimized techniques to enhance heat transfer [1,2]. The nanofluid concept was pioneered by Choi in 1995 [2], which are stable colloidal dispersions of solid nanoparticles (typically with sizes in the range of 1–100 nm) in base fluids, compared to traditional base fluids such as water, ethylene glycol, oil, and so on. Nanofluids have many excellent properties due to the large specific surface of nanoparticles. Nanoparticles are expected to greatly enhance the thermal conductivity and improve the stability of nanofluids due to their unique properties [3,4]. Consequently, many scientists have conducted substantial and theoretical studies on the different respects of thermal conductivity of nanofluids with various nanoparticles. The hypothetical nanomaterial candidates for nanofluid formulations are metals, metal oxides, and carbon materials. In the past decade, many kinds of the above materials have been studied to produce nanofluids, such as Ag, Au, CuO, Al_2_O_3_, ZnO, and carbon nanotubes [5]. Patel el al. [6] reported an enhancement in thermal conductivity of about 5–21% for an Au nanofluid at a loading volume fraction of 0.00026% in the temperature range of 30–60 °C, compared to deionized (DI)-water. Zhao et al. [4] found thermal conductivity could be enhanced with an increase of the Al_2_O_3_ nanoparticle volume fraction and temperature, with a maximum enhancement of 28% obtained at a nanoparticle volume fraction of 5.92% and a temperature of 313 K. Chandrasekar et al. [7] experimentally investigated the effective thermal conductivities and viscosities of water-based nanofluids containing Al_2_O_3_ nanoparticles. Karthikeyan et al. [8] found an ethylene glycol-based CuO nanofluid with a 1% volume fraction gave a 54% enhancement in the thermal conductivity. It was reported that the convective heat transfer coefficient of functionalized MWNT nanofluids was enhanced by up to 33–40% at a concentration of 0.25 wt %, compared with that of pure water in laminar and turbulent flows, respectively, at 20 °C [9]. 

Recently, graphene nanofluids have attracted many researchers’ attentions owing to their variety of remarkable properties, including high thermal conductivities, extraordinary electronic transport property, large specific surface areas, and so on [10,11,12]. Outstanding thermo-physical characteristic of graphene has made it a potential candidate in the heat-transfer fluid field. The investigations have indicated that graphene-based nanofluids have higher heat transfer and thermal conductivity properties than other carbon materials [10,13,14,15,16]. Tessy et al. [10] reported an enhancement in thermal conductivity of water-based graphene nanofluids with a very low volume fraction of 0.056% by about 14% at 25 °C, which increases to about 64% at 50 °C. A substantial thermal conductivity enhancement of graphene nanofluids was obtained even at lower concentrations, the enhancement increased from 10% to 27% with the temperature increasing from 20 to 50 °C at 0.2 vol % concentration [13]. The enhancement also shows strong temperature dependence. The alkaline-functionalized graphene nanofluid also showed good thermal conductivity with the enhancement around 14.1% at 25 °C and 17% at 50 °C compared to water [15]. Nevertheless, due to the high naturally-hydrophobic character of graphene, it cannot be dispersed in any polar solvent, such as ethylene glycol and water. In addition, the van der Waals force and the strong π-π interactions between the planar basal planes give rise to the graphene nanosheets to restack themselves and easily agglomerate in aqueous solution [17]. Therefore, it is much more difficult for graphene to be dispersed in water and suspended in a stable manner. Adding surfactants is considered to be the simplest way to avoid sediment and improve the stability of graphene nanofluids [18,19]. However, low thermal conductivity of the surfactants may decrease the heat-transfer characteristics of the nanofluids [20]. Meanwhile, strong oxidants have been applied successfully to introduce hydrophilic hydroxyl and carboxyl functional groups into graphene to make graphene more hydrophilic [21,22,23,24]. However, the structure of graphene will be destroyed during the process of acid treatment, and lead to a reduction of thermal conductivity [15,25]. Recent studies demonstrate that graphene with metal oxide nanocomposites may be one of the effective approaches to solve such problems. Due to the synergistic effect, the metal oxides could not only prevent the graphene nanosheets from restacking, but also improve thermal conductivity of the nanofluids. Mohammad et al. [26] prepared a rGO-Fe_3_O_4_ nanofluid with high stability. The thermal conductivity was enhanced up to 11% at the mass fraction of 0.5%. Hooman et al. [27] studied the functionalized graphene nanoplatelet nanofluid, it was stable and no sedimentation was observed for a long time. The enhancement of thermal conductivity was 16.94% at 20 °C and nearly 22.22% at 40 °C for 0.1% weight concentration. Baby and Sundara reported the enhancement value of thermal conductivities for CuO decorated graphene dispersed nanofluid containing a volume fraction of 0.05% at 25 °C could reach about 28% [28]. Li et al. [29] found better thermal conductivity and stability for SiO_2_-coated graphene nanofluids, compared to graphene fluids. Wang et al. [30] reported the water-based TiO_2_ anchored graphene nanofluids have good dispersion stability and the maximum value of thermal conductivity enhancement was up to 33% at a mass fraction of 0.1%. 

On the other hand, SnO_2_ water-based nanofluids show a good enhancement of thermal conductivity as well [31,32]. Habibzadeh et al. [31] found that the thermal conductivity of water-based SnO_2_ nanofluids at a weight fraction of 0.024% was enhanced up to 8.7%. The electrochemical performance, specific capacitance, and cycling stability of SnO_2_/rGO nanocomposite have been investigated as well [33,34,35]. However, to the best of our knowledge, there are few reports concentrating on the thermal conductivity of SnO_2_/rGO nanocomposite dispersed nanofluids. In this work, a novel tin oxide/graphene oxide nanocomposite was synthesized by the solvothermal process. The thermal conductivity properties and stability of water-based nanofluids dispersed by the hybrid nanocomposite with various concentrations was investigated in detail. 

## 2. Experimental

### 2.1. Materials

All of the reagents used in the experiments were of analytical grade and used without further purification. Graphene oxide (GO) with purity ~99% maximum particle diameter of 3 µm and maximum thickness of 1.2 nm was purchased from Chengdu Organic Chemicals Co., Ltd. (Chengdu, China), Chinese Academy of Sciences. Tin chloride pentahydrate (SnCl_4_·5H_2_O, 99.0%) and absolute ethanol (99.8%) were purchased from Sigma-Aldrich (St. Louis, MO, USA).

### 2.2. Synthesis of the SnO_2_/rGO Nanocomposites

The SnO_2_/rGO hybrid composites were prepared by a simple hydrothermal method. Tin chloride pentahydrate (SnCl_4_·5H_2_O) (0.1 M) was dissolved in 56 mL absolute ethanol under continuous magnetic stirring and then treated by ultrasonication for 15 min. Then 4 mL GO aqueous solution (2.5 g/mL) was dropped into the above mixture slowly under magnetic stirring. After several minutes, the mixture was transferred into a stainless steel autoclave and kept at 120 °C for 6 h to synthesize tin oxide/reduced graphene oxide composites. The black product was centrifuged and washed several times with deionized water and absolute ethanol, and then dried at 60 °C for 12 h. For comparison, the reduced graphene oxide (rGO) and pure SnO_2_ were also prepared via the same process without adding SnCl_4_ and GO, respectively.

### 2.3. Characterization of the SnO_2_/rGO Nanocomposites

X-ray diffraction (XRD) was employed to identify the crystalline structure of the gained samples with an X-ray powder diffractometer (Cuk_α_, DMAX-UltimaIV, Rigaku Co., Tokyo, Japan). A transmission electron microscope (TEM, JEM-2100F, JEOL, Tokyo, Japan) was used to study the detailed microstructures and morphology of the samples. An X-ray photoelectron spectroscope (XPS, Kratos AXIS ULTRA DLD, Shimadzu, Kyoto, Japan) was operated to investigate the oxidation states of Sn and graphene, as well as the elemental composition of the SnO_2_/rGO nanocomposite. Thermogravimetric analysis (TGA, SDT-2960, TA Instruments, New Castle, DE, USA) measurements were performed to analyze the quality percentage of SnO_2_ in the SnO_2_/rGO compound, the samples were heated from room temperature to 900 °C at the rate of 10 °C/min in the air. The molecular vibration mode and defects of the samples were analyzed by Raman spectroscopy (RM2000, Renishaw, London, UK). Fourier transform infrared spectroscopy (FTIR, Nicolet iz10, Thermol scientific, Waltham, MA, USA) was used to analyze and identify the functional groups on the surface of the samples. 

### 2.4. Preparation and Property Measurement of Water-Based Nanofluids

A high-power ultrasonication probe (HNF2000, Huanan Ultrasonic Equipment Co., Ltd., Guangzhou, China) which supplies 2000 W output power and a 20-khz frequency was used to prepare the SnO_2_/rGO hybrid water-based nanofluids. SnO_2_/rGO nanocomposite was dispersed in DI water to prepare nanofluids with different weight concentrations, including 0.02, 0.04, 0.06, 0.08, and 0.1 wt %, without adding any surfactants. As a comparison, pure SnO_2_ and rGO water-based nanofluids were also prepared using the same process, respectively. Zeta potential testing was employed to assess the dispersion stability of above nanofluids. A transient heated needle (KD2, Decagon, Device, TPS 500s, Hot Disk, Uppsala, Sweden) with 5% accuracy was used to measure the thermal conductivity of nanofluids based on the transient hot-wire method (THW). To avoid enhancement of effective viscosity of nanofluids, the low weight concentrations of as-prepared nanofluids in the range of 0.02–0.1 wt % were chosen, and the temperature is between from 20 to 60 °C. Each sample was repeated five times with each measurement interval of 30 min, and the average value of thermal conductivity was obtained.

## 3. Results and Discussion

### 3.1. Characterization of Prepared SnO_2_/rGO Nanocomposites

**Figure 1:**
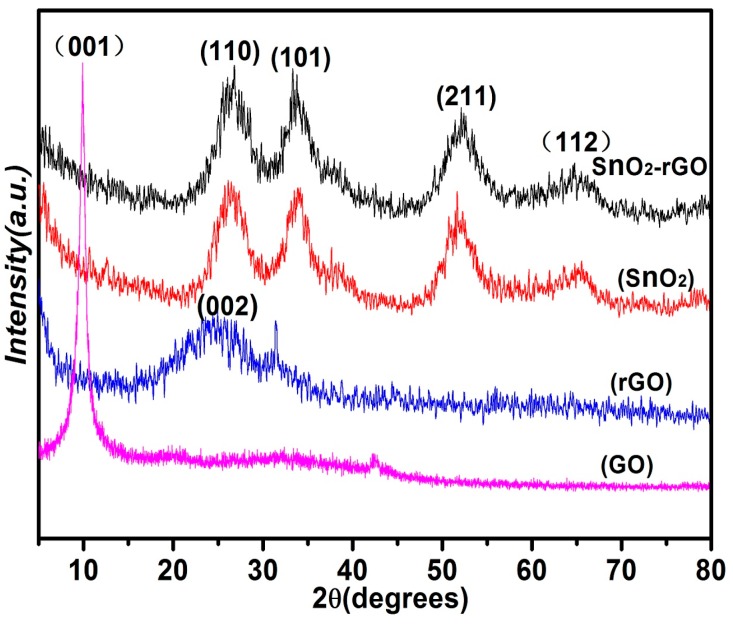
X-ray diffraction of GO, rGO, SnO_2_, and SnO_2_/rGO nanocomposites.

The structure information and crystal phase of the samples were investigated by X-ray diffraction (XRD) analysis. Figure 1 shows the XRD patterns of GO, rGO, Pure SnO_2_, and SnO_2_/rGO nanocomposite samples. The diffraction peak observed at 2θ = 10.5° is corresponding to (001) characteristic peak of GO. A broad diffraction peak centered at 25.1° appears, which can contribute to the (002) plane diffraction of rGO sheets, indicating a poor degree of graphite-like material [36]. This result reveals that the GO has been reduced into rGO through the decomposition of oxygenated functionalities during the solvothermal process and the few-layer structure of rGO has been formed. It is clear that the XRD patterns of pure SnO_2_ and SnO_2_/rGO nanocomposite are basically the same. The diffraction peaks at 2θ = 26.5°, 33.8°, 51.7°, and 64.7° could correspond to the (110), (101), (211), and (112) crystalline planes of SnO_2_ (JCPDS no. 41-1445), respectively [37]. The average size of particles calculated by the Debye-Scherrer equation is 4.5 nm, indicating the successful formation of ultra-small SnO_2_ nanocrystals. 

**Figure 2:**
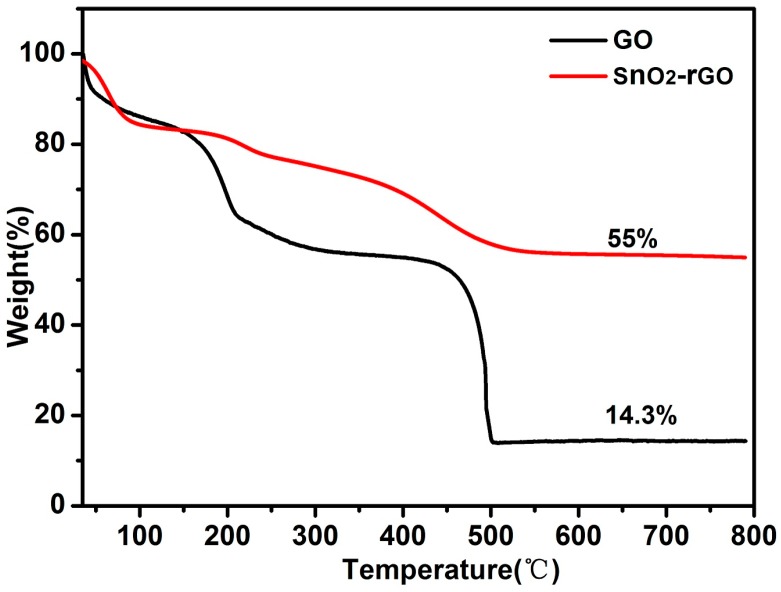
Thermogravimetric curves of SnO_2_/rGO compound and GO.

Thermogravimetric analysis (TGA, SDT-2960, TA Instruments, New Castle, DE, USA) measurements were performed to analysis the quality percentage of SnO_2_ in the SnO_2_/rGO compound. As shown in Figure 2, there are two obvious weight loss processes for GO and SnO_2_/rGO. The weight loss in the range of room temperature to 150 °C is owing to the dislodgement of absorbed water and carbon combustion. The mass loss from 150 to 400 °C corresponds to the decomposition of oxygen-containing groups. At high temperatures, from 400 to 500 °C, the weight loss is attributed to the destruction of the carbon skeleton. This decomposing process causes significant weight loss, indicating that there is no GO or rGO remaining in the samples [35]. The residual mass fraction for the GO sample still reaches 14.3%, which is probably due to the introduction of impurities during preparation process. As for the SnO_2_/rGO compound, the mass loss equals to 45% in the range of room temperature to 500 °C. That is, the remaining weight percentage of the hybrid sample stays 55%. Therefore, according to the TGA results, the calculated weight percentage of SnO_2_ is about 40% in the SnO_2_/rGO composites.

**Figure 3:**
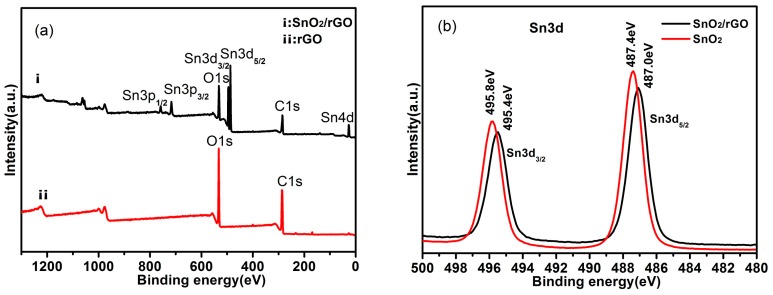

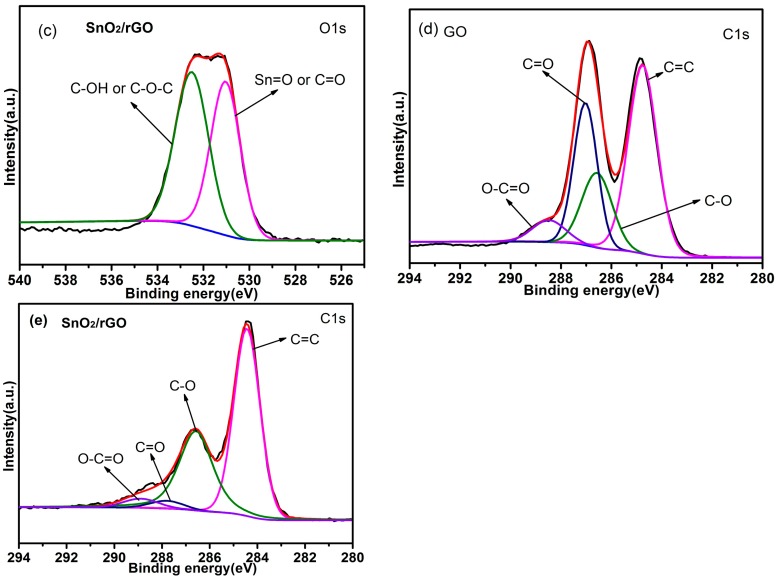
XPS of GO and SnO_2_/rGO composite. (**a**) XPS survey spectra of GO and SnO_2_/rGO; (**b**) XPS spectrum of Sn3d of SnO_2_/rGO and SnO_2_; (**c**) O 1s spectra of SnO_2_/rGO; and (**d**,**e**) C1s spectra of GO and SnO_2_/rGO.

XPS is a forceful tool to investigate the surface chemistry of the samples. As shown in Figure 3a, the as-prepared SnO_2_/rGO nanoparticles contain C, O, and Sn elements. In the core-level XPS signals of Sn3d (Figure 3b), the Sn3d_3/2_ and Sn3d_5/2_ peaks are observed at 495.8 and 487.4 eV, respectively, corresponding to Sn^4+^ ions in the tetragonal rutile structure of SnO_2_ [38]. For the SnO_2_/rGO nanocomposites, the shift of 0.4 eV is observed for the Sn3d_3/2_ and Sn3d_5_/_2_ peaks, which may be due to the reaction of Sn^4+^ ions with the active sites of rGO. Figure 3c displays the O1s XPS spectrum of the SnO_2_/rGO nanocomposites can be divided into two peaks. One is at the binding energy of 531 eV, which corresponds to the C=O or Sn=O group of SnO_2_. Another peak at 532.5 eV is attributed to the C–OH and C–O–C groups. The above results verify that the Sn element exists in the compound state of SnO_2_. C1s core-level XPS signals of GO and SnO_2_/rGO can be deconvoluted into four components, including C–C/C=C, C–O, C=O carbonyl and carboxylic groups, and O–C=O carboxylate carbon groups, respectively, as shown in Figure 3d,e [39]. The peaks of oxygen-containing groups for SnO_2_/rGO have been greatly suppressed compared with GO, which has abundant oxygenated groups. This demonstrates that the majority of oxygenated functional groups have been removed after the solvothermal process. However, a small amount of residual oxygenated groups still remain, indicating that GO has been partially reduced. These hydrophilic functional groups are advantageous for SnO_2_/rGO to be stably dispersed in aqueous solution for a long time.

**Figure 4:**
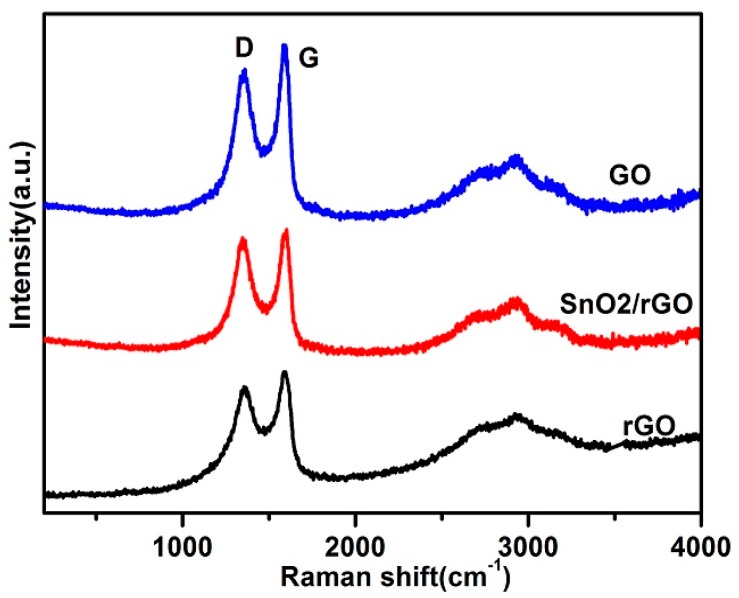
Raman spectra of GO, rGO, and SnO_2_/rGO.

The structure disordered degree of the carbonaceous materials can be gained by Raman spectroscopy. It is obvious that there are two prominent peaks at around 1351 and 1591 cm^−1^, corresponding to the D-band for carbon structure and the G-band for defects, respectively, as shown in Figure 4. Attributed to the solvothermal reduction of GO, the peak position is shifted which shows the damage of rGO network and the formation of defects [29]. Moreover, the intensity ratio of the D to G bands (I_D_/I_G_) normally indicates the defect degree of the materials. The I_D_/I_G_ for GO, rGO, and SnO_2_/rGO composite are 0.88, 0.91, and 0.96, respectively, which means rGO in the SnO_2_/rGO nanocomposites has more defects compared with the pure rGO, revealing the SnO_2_ nanoparticles intercalating into the rGO sheets. 

**Figure 5:**
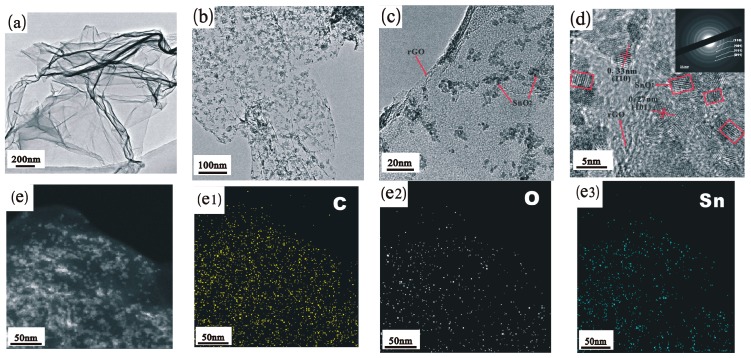
(**a**) TEM images of rGO; (**b**,**c**) TEM images of SnO_2_/rGO under different magnification; (**d**) HRTEM images of SnO_2_/rGO, and the inset is the corresponding SAED pattern; and (**e**) elemental mapping of SnO_2_/rGO depicting the even distribution of C (**e1**), O (**e2**), and Sn (**e3**).

Transmission electron microscopy (TEM) has been employed to further characterize the morphologies and crystal structure of the SnO_2_/rGO composite. Figure 5a shows the rGO sheets are almost transparent with some wrinkles on the surface and folding at the edges under TEM. For the SnO_2_/rGO nanocomposite, as shown in Figure 5b,c, numerous nanoparticles are uniformly distributed on the wrinkled rGO nanosheets with the average particle size from 3 to 5 nm. It is consistent with the result we calculated from XRD. High-resolution TEM imagery (Figure 5d) reveals that the nanoparticles are crystalline, having a calculated lattice spacing of 0.33 and 0.27 nm, which are consistent with the (110) and (101) planes of rutile SnO_2_ [37]. The corresponding SAED pattern (inset of Figure 5d) further confirms the presence of the (110), (101), (111), and (211) lattice planes of SnO_2_ (JCPDS.no. 41-1445) [40,41]. Those results are also in good agreement with the XRD results, indicating SnO_2_ nanoparticles have been synthesized on the surface of rGO. Elemental mapping results confirm that the ultrasmall SnO_2_ nanoparticles dispersed homogenously as well. As illustrated in Figure 5e, the C, O, and Sn elements distribute uniformly in the selected area with high density. Considering the ultrasonic process used during the sample preparation for TEM observation, these results clearly demonstrate that the SnO_2_ nanoparticles have been successfully prepared and are anchored firmly on the rGO surface with high packing density. 

### 3.2. Stability of the Water-Based SnO_2_/rGO Nanofluids

**Figure 6:**
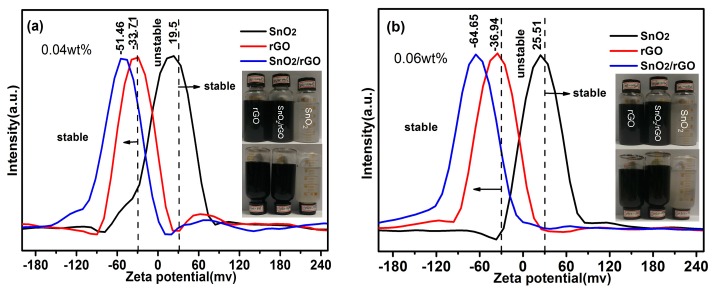
Zeta potential value of SnO_2_/rGO composite, rGO, and SnO_2_ nanofluids with different concentration; (**a**) 0.04 wt % and (**b**) 0.06% insets are photographs of rGO, SnO_2_/rGO, and SnO_2_ nanofluids after quiescence for 30 days.

Zeta potential measurements and the sedimentation have been applied to identify the stability of the SnO_2_/rGO nanofluids as they are common and important methods to evaluate the dispersion behavior of nanoparticles in a liquid environment. In general, the nanofluid is referred to have good stability when the zeta potential values are higher than 30 mV [16]. The zeta potential values of rGO, SnO_2_, and SnO_2_/rGO nanofluids with mass fractions of 0.04% and 0.06% are shown in Figure 6. It is clear that the absolute zeta potential values of both rGO and SnO_2_/rGO nanofluids are higher than 30 mV, in which SnO_2_/rGO nanofluids has higher zeta potential value (above 50 mV) than the rGO nanofluids (about 33 mV). However, for SnO_2_ nanofluid, the value is less than 30 mV implying poor dispersion stability. The results indicate the suspension stability of SnO_2_/rGO nanofluid is better than the rGO and SnO_2_ nanofluids. The sedimentation photographs of the nanofluids after quiescence for 30 days also clearly show that only a small amount of precipitation could be observed for rGO and SnO_2_/rGO nanofluids, and SnO_2_ nanofluids present a pronounced precipitation over time, as exhibited in insets of Figure 6. This phenomenon may be due to the fact that on the surface of rGO still remain a small amount of hydrophilic functional groups, such as hydroxyl, carboxyl, and carbonyl groups, after the hydrothermal reaction process. Furthermore, as the ultrasmall SnO_2_ nanoparticles are decorated on the surface of reduced graphene oxide, the graphene sheets could avoid overlapping or stacking. Hence, this ensures the large specific surface area of rGO, good stability, and thermal conductivity for the nanofluids. 

**Figure 7:**
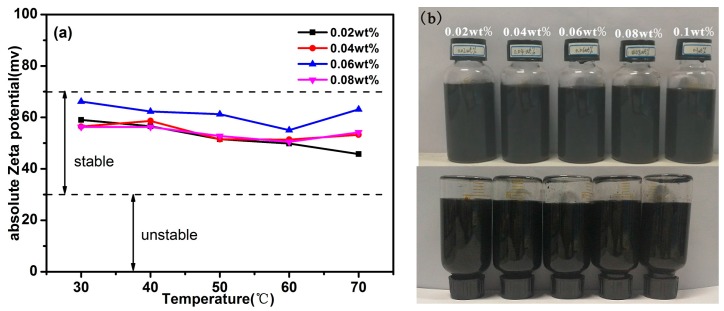
(**a**) Effect of temperature on the absolute Zeta potential values at different concentrations; (**b**) Photographs of 0.02, 0.04, 0.06, 0.08, and 0.1 wt % water-based nanofluids of SnO_2_/rGO after quiescence for 60 days.

Temperature and concentration play an important role on the stability of nanofluids. As plotted in Figure 7a, it is obvious that the absolute zeta potential values of the SnO_2_/rGO nanofluids with mass fractions of 0.02%, 0.04%, 0.06%, and 0.08% are all higher than 40 mV at temperatures from 30 to 70 °C. This indicates that the prepared SnO_2_/rGO nanofluids possess much better dispersion stability. Sedimentation observation of the nanofluids after quiescence for 60 days demonstrate clearly that the SnO_2_/rGO nanofluids exhibit good long time dispersion stability for each prepared concentration, which also coincide with zeta potential values, as shown in Figure 7b. No obvious sedimentation is observed for all nanofluids samples, whereas little agglomeration and sedimentation are found in the samples with higher concentration, especially at 0.1 wt %. 

### 3.3. Thermal Conductivity of the Water-Based SnO_2_/rGO Nanofluids

**Figure 8:**
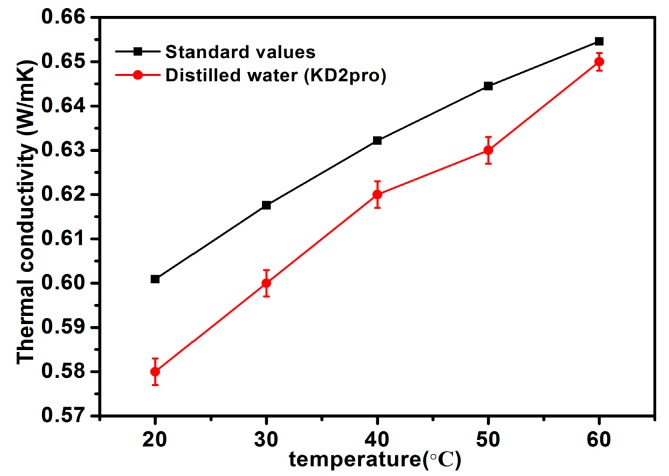
Comparison of the measured values of thermal conductivity using KD2 Pro for distilled water with the standard values presented by Ramires [42].

Thermal conductivity is one of the most important values for nanofluids. In this work, the thermal properties analyzer (KD2 pro, Decagon, Device, TPS 500s, Hot Disk, Uppsala, Sweden) was employed to measure the thermal conductivity of nanofluids which has a 5% accuracy. The theory of measurement was based on the transient hot-wire method (THW). Before measuring the thermal conductivity of nanofluids, the thermal conductivity of deionized water was measured at 20–60 °C to calibrate the experimental apparatus, using the standard thermal conductivity value of deionized water which comes from Ramires et al. [42]. It is clear the results are gained a maximum of error 3.48%, as demonstrated in Figure 8. Therefore, it can be concluded that the KD2 Pro worked within its designed accuracy. In order to avoid enhancement of effective viscosity of nanofluids, the low weight concentrations of the as-prepared nanofluids in the range of 0.02–0.1 wt % were chosen, and the temperature was set between 20 and 60 °C. Each sample was repeated five times with each measurement interval of 30 min, and the average value of thermal conductivity was obtained. 

**Figure 9:**
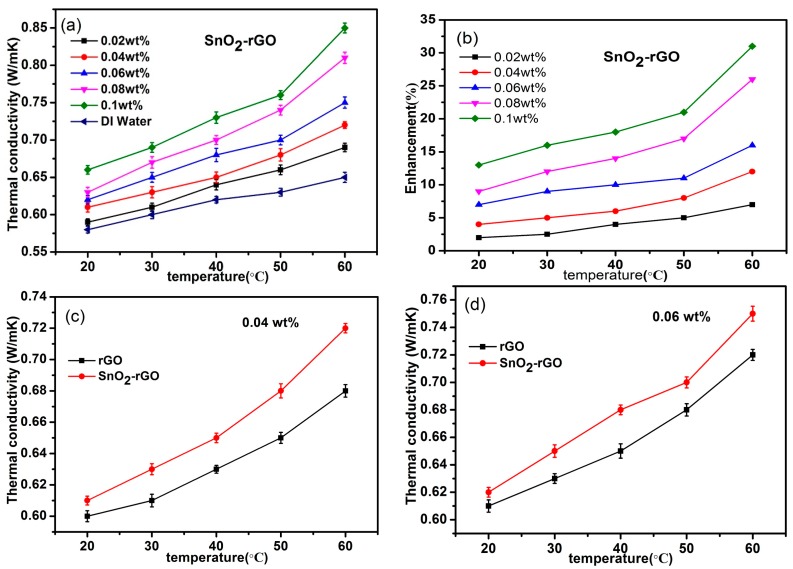
Thermal conductivity of water-based rGO and SnO_2_/rGO nanofluids as a function of temperature and the weight fraction of nanoparticles. (**a**) SnO_2_/rGO water-based nanofluids at different concentrations; (**b**) Effect of temperature on the thermal conductivity enhancements at different concentrations; (**c**,**d**) Comparison of SnO_2_/rGO and rGO water-based nanofluids at different concentrations of 0.04 wt % and 0.06 wt %.

The thermal conductivity properties of water-based SnO_2_/rGO nanofluids at different weight concentrations as a function of temperature are shown in Figure 9. The thermal conductivity of deionized water and water-based pure rGO nanofluids with the same mass fraction have been measured as well for comparison. It is obvious that the thermal conductivity of SnO_2_/rGO nanofluids are higher than that of deionized water. The percentage enhancement in thermal conductivity (K_eff_) is calculated through the formula ((K − K_0_) × 100%)/K_0_, where K_0_ and K represents the thermal conductivity of the base fluid (deionized water) and nanofluid, respectively. As shown in Figure 9a,b, the mass fraction and temperature have significant influence on the thermal conductivity of SnO_2_/rGO nanofluids. The thermal conductivities of nanofluids enhance at various degrees as concentrations of SnO_2_/rGO nanocompounds and temperatures increase. There are several possible suggested mechanisms to explain the enhancement of thermal conductivity for nanofluids [43,44]. When the temperature of nanofluids increases, the Brownian motion of nanoparticles enhances which leads to higher thermal conductivity of nanofluids. With the increasing of nanoparticles weight concentration, the distance between particles (free path) decreases. More nanoparticles are in contact with each other, resulting in the frequency of the lattice vibration increasing and the heat transfer of electrons and phonons improving [28]. As a result, the higher the concentration of nanoparticles, the higher the thermal conductivity, which is consistent with Maxwell’s theory [45]. In addition, it is well known that there is a nano-layered structure at the solid-liquid interface when the liquid molecules are close to the solid surface. This solid-like nano-layer structure acts as a medium of heat transport from the solid to the bulk liquid which may be a major contributing mechanism to the enhancement of thermal conductivity in the nanofluid [46]. When the temperature rises from 20 to 60 °C, the enhancement of thermal conductivity icnreases from 2% to 7% at a mass fraction of 0.02 wt %, and reaches 7% to 16% at a mass fraction of 0.06 wt %. The maximum enhancement of thermal conductivity approaches to 31% at 60 °C for the concentration of 0.1 wt %. This phenomenon of non-linear enhancement is consistent with others’ previous studies [10,30]. It can be seen clearly from the results displayed in Figure 9c,d that the thermal conductivities of SnO_2_/rGO nanofluids are higher than rGO nanofluids under various mass fractions. It is suggested that SnO_2_ nanoparticles are decorated on the surface of rGO, which can block the stacking of graphene sheets. As a result, water-based SnO_2_/rGO nanofluids have better stability and exhibit better thermal conductivity. 

It is evident that the thermal conductivity of the nanofluids relies on temperature and concentration when the nanoparticles and the base fluid are assigned. Consequently, it is clear that the thermal conductivity and dimension of the solid-liquid interfacial layer have significant impacts on the enhanced thermal conductivity of nanofluids. The typical theoretical models that have been developed for thermal conductivity of nanoparticles suspended fluids only consider thermal conductivities of the base fluid, particles, and volume fraction of particles, while particle size, shape, the distribution and motion of dispersed particles and micro convection are having important influences on thermal conductivity enhancement as well [47]. Therefore, the experimental results sometime could not be compared with the correlated values of the theoretical models [30,48]. Furthermore, the comparison between graphene-based nanofluids in most recent works is shown in Table 1, it is apparent that water-based SnO_2_/rGO nanofluids with low additive concentration possess a pretty good thermal conductivity enhancement, compared to those of other works with higher concentration of nanoparticles. From above results, it can be concluded that the SnO_2_/rGO nanocomposite could possibly be a useful candidate obtain satisfactory thermal conductivity enhancement for medium-temperature coolant applications.

## 4. Conclusions

In this paper, SnO_2_/rGO nanocomposites with ultrasmall-sized SnO_2_ nanoparticles anchored on the surface of rGO have been synthesized successfully via a simple solvothermal reaction. The well-dispersed water-based SnO_2_/rGO nanofluids with different weight concentrations have been prepared without any surfactant as well. The results reveal that the SnO_2_/rGO nanofluids have good dispersion stability. The absolute values of zeta potential for water-based SnO_2_/rGO nanofluids are basically high than 30 mV and only a few sedimentation can be observed after quiescence for 60 days. The thermal conductivity of SnO_2_/rGO nanofluids increases with increasing temperature, as well as weight concentration. The enhancement of thermal conductivity of water-based SnO_2_/rGO nanofluids could reach up to 31% at 60 °C under the mass fraction of 0.1 wt %, comparing to deionized water. The SnO_2_/rGO nanofluids also possess higher thermal conductivity than the water-based rGO nanofluid. The above results show the water-based SnO_2_/rGO nanofluids have a bright prospect in the industrial application of heat exchangers systems. However, the electrical conductivity, viscosity, and convective heat transfer coefficient of nanofluids have not yet been studied widely compared to their thermal conductivity, which are important for nanofluid research. Additionally, investigations about theoretical models of the mechanism of thermal conductivity and heat exchange enhancement are needed for further study. 

## Figures and Tables

**Table 1:** Comparison of thermal conductivity enhancements of our as-prepared nanofluid with reported graphene-based nanofluids.

Materials	Base Fluid	Loading	Enhancement %	References
graphene	water	0.2 vol %	27	[13]
graphene	water	0.05 wt %	17	[15]
rGO	water	0.03 wt %	10	[16]
f-HEG	water	0.05 vol %	16	[23]
rGO-Fe_3_O_4_	water	0.5 vol %	11	[26]
GNP–Ag	water	0.1 wt %	22	[27]
graphene-CuO	water	0.5 vol %	28	[28]
graphene-SiO_2_	water	0.1 wt %	20	[29]
rGO/TiO_2_	water	0.1 wt %	33	[30]
SnO_2_/rGO	water	0.1 wt % (0.078 vol %)	31	This work

## References

[1] Kasaeian A., Daneshazarian R., Mahian O., Kolsi L., Chamkha A.J., Wongwises S., Pop I. (2017). Nanofluid flow and heat transfer in porous media: A review of the latest developments. Int. J. Heat Mass Transf..

[2] Eggers J.R., Kabelac S. (2016). Nanofluids revisited. Appl. Therm. Eng..

[3] Agromayor R., Cabaleiro D., Pardinas A.A., Vallejo J.P., Fernandez-Seara J., Lugo L. (2016). Heat transfer performance of functionalized graphene nanoplatelet aqueous nanofluids. Materials.

[4] Zhao N., Li Z. (2017). Experiment and artificial neural network prediction of thermal conductivity and viscosity for alumina-water nanofluids. Materials.

[5] Devendiran D.K., Amirtham V.A. (2016). A review on preparation, characterization, properties and applications of nanofluids. Renew. Sustain. Energy Rev..

[6] Patel H.E., Das S.K., Sundararajan T., Sreekumaran N.A., George B., Pradeep T. (2003). Thermal conductivities of naked and monolayer protected metal nanoparticle based nanofluids: Manifestation of anomalous enhancement and chemical effects. Appl. Phys. Lett..

[7] Chandrasekar M., Suresh S., Chandra B.A. (2010). Experimental investigations and theoretical determination of thermal conductivity and viscosity of Al_2_O_3_/water nanofluid. Exp. Therm. Fluid Sci..

[8] Karthikeyan N.R., Philip J., Raj B. (2008). Effect of clustering on the thermal conductivity of nanofluids. Mater. Chem. Phys..

[9] Amrollahi A., Rashidi A.M., Lotfi R., Emami M.M., Kashefi K. (2010). Convection heat transfer of functionalized mwnt in aqueous fluids in laminar and turbulent flow at the entrance region. Int. Commun. Heat Mass Transf..

[10] Baby T.T., Ramaprabhu S. (2010). Investigation of thermal and electrical conductivity of graphene based nanofluids. J. Appl. Phys..

[11] Marcano D.C., Dmitry V.K., Berlin J.M., Sinitskii A., Sun Z., Slesarev A., Alemany L.B., Lu W., Tour J.M. (2010). Improved synthesis of graphene oxide. ACS Nano.

[12] Aravind S.S.J., Ramaprabhu S. (2013). Graphene-multiwalled carbon nanotube-based nanofluids for improved heat dissipation. RSC Adv..

[13] Sen G.S., Manoj S.V., Krishnan S., Sreeprasad T.S., Singh P.K., Pradeep T., Das S.K. (2011). Thermal conductivity enhancement of nanofluids containing graphene nanosheets. J. Appl. Phys..

[14] Kole M., Dey T.K. (2013). Investigation of thermal conductivity, viscosity, and electrical conductivity of graphene based nanofluids. J. Appl. Phys..

[15] Ghozatloo A., Shariaty-Niasar M., Rashidi A.M. (2013). Preparation of nanofluids from functionalized graphene by new alkaline method and study on the thermal conductivity and stability. Int. Commun. Heat Mass Transf..

[16] Kamatchi R., Venkatachalapathy S., Abhinaya S.B. (2015). Synthesis, stability, transport properties, and surface wettability of reduced graphene oxide/water nanofluids. Int. J. Therm. Sci..

[17] Liao Q., Li N., Jin S., Yang G., Wang C. (2015). All-solid-state symmetric supercapacitor based on Co_3_O_4_ nanoparticles on vertically aligned graphene. ACS Nano.

[18] Sarsama W.S., Amiri A., Kazi S.N., Badarudin A. (2016). Stability and thermophysical properties of non-covalently functionalized graphene nanoplatelets nanofluid. Energy Convers. Manag..

[19] Uddin M.E., Kuila T., Nayak G.C., Kim N.H., Ku B.-C., Lee J.H. (2013). Effects of various surfactants on the dispersion stability and electrical conductivity of surface modified graphene. J. Alloys Compd..

[20] Leong K., Mohd N.H., Mohd S., Amer N. (2016). The effect of surfactant on stability and thermal conductivity of carbon nanotube based nanofluids. Therm. Sci..

[21] Lin Y., Zhang H., He C., Li Y., Wang S., Hong H. (2017). A new kind of water-based nanofluid with a low loading of three-dimensional porous graphene. J. Mater. Sci..

[22] Tchoul M.N., Ford W.T., Lolli G., Resasco D.E., Arepalli S. (2007). Effect of mild nitric acid oxidation on dispersability, size, and structure of single-walled carbon nanotubes. Chem. Mater..

[23] Baby T.T., Ramaprabhu S. (2011). Enhanced convective heat transfer using graphene dispersed nanofluids. Nanoscale Res. Lett..

[24] Liu M., Yang Y., Zhu T., Liu Z. (2005). Chemical modification of single-walled carbon nanotubes with peroxytrifluoroacetic acid. Carbon.

[25] Zhang L., Ni Q., Fu Y., Natsukia T. (2009). One-step preparation of water-soluble single-walled carbon nanotubes. Appl. Surf. Sci..

[26] Mehrali M., Sadeghinezhad E., Akhiani A.R., Tahan L.S., Metselaar H.S.C., Kherbeet A.S., Mehrali M. (2017). Heat transfer and entropy generation analysis of hybrid graphene/Fe_3_O_4_ ferro-nanofluid flow under the influence of a magnetic field. Powder Technol..

[27] Yarmand H., Gharehkhani S., Ahmadi G., Shirazi S.F.S., Baradaran S., Montazer E., Zubir M.N.M., Alehashem M.S., Kazi S.N., Dahari M. (2015). Graphene nanoplatelets-silver hybrid nanofluids for enhanced heat transfer. Energy Convers. Manag..

[28] Baby T.T., Sundara R. (2011). Synthesis and transport properties of metal oxide decorated graphene dispersed nanofluids. J. Phys. Chem. C.

[29] Li X., Chen Y., Mo S., Jia L., Shao X. (2014). Effect of surface modification on the stability and thermal conductivity of water-based SiO_2_-coated graphene nanofluid. Thermochim. Acta.

[30] Wang S., Li Y., Zhang H., Lin Y., Li Z., Wang W., Wu Q., Qian Y., Hong H., Zhi C. (2016). Enhancement of thermal conductivity in water-based nanofluids employing TiO_2_/reduced graphene oxide composites. J. Mater. Sci..

[31] Habibzadeh S., Kazemi-Beydokhti A., Khodadadi A.A., Mortazavi Y., Omanovic S., Shariat-Niassar M. (2010). Stability and thermal conductivity of nanofluids of tin dioxide synthesized via microwave-induced combustion route. Chem. Eng. J..

[32] Mariano A., Pastoriza-Gallego M.J., Lugo L., Camacho A., Canzonieri S., Piñeiro M.M. (2013). Thermal conductivity, rheological behaviour and density of non-newtonian ethylene glycol-based SnO_2_ nanofluids. Fluid Phase Equilib..

[33] Zhang D., Liu J., Chang H., Liu A., Xia B. (2015). Characterization of a hybrid composite of SnO_2_ nanocrystal-decorated reduced graphene oxide for ppm-level ethanol gas sensing application. RSC Adv..

[34] Jiang S., Yue W., Gao Z., Ren Y., Ma H., Zhao X., Liu Y., Yang X. (2013). Graphene-encapsulated mesoporous SnO_2_ composites as high performance anodes for lithium-ion batteries. J. Mater. Sci..

[35] Chen L., Ma X., Wang M., Chen C., Ge X. (2016). Hierarchical porous SnO_2_ /reduced graphene oxide composites for high-performance lithium-ion battery anodes. Electrochim. Acta.

[36] Xu C., Wang X., Zhu J. (2008). Graphene-metal particle nanocomposites. J. Phys. Chem..

[37] Wang Y., Ding J., Liu Y., Liu Y., Cai Q., Zhang J. (2015). SnO_2_@reduced graphene oxide composite for high performance lithium-ion battery. Ceram. Int..

[38] Zhang X., Huang X., Zhang X., Xia L., Zhong B., Zhang T., Wen G. (2017). Cotton/rGO/carbon-coated SnO_2_ nanoparticle-composites as superior anode for lithium ion battery. Mater. Des..

[39] Gu Y., Xing M., Zhang J. (2014). Synthesis and photocatalytic activity of graphene based doped TiO_2_ nanocomposites. Appl. Surf. Sci..

[40] Li F., Song J., Yang H., Gan S., Zhang Q., Han D., Ivaska A., Niu L. (2009). One-step synthesis of graphene/sno_2_ nanocomposites and its application in electrochemical supercapacitors. Nanotechnology.

[41] Wu Q.-H., Wang C., Ren J.-G. (2013). Sn and SnO_2_-graphene composites as anode materials for lithium-ion batteries. Ionics.

[42] Ramires M.L.V., de Castro C.A.N. (1995). Standard reference data for the thermal conductivity of water. J. Phys. Chem. Ref. Data.

[43] María J.P.-G., Luis L., Legido J.L., Piñeiro M.M. (2011). Thermal conductivity and viscosity measurements of ethylene glycol-based Al_2_O_3_ nanofluids. Nanoscale Res. Lett..

[44] Keblinski P., Phillpot S.R., Choi S.U.S., Eastman J.A. (2002). Mechanisms of heat flow in suspensions of nano-sized particles (nanofluids). Int. J. Heat Mass Transf..

[45] Yu W., Choi S.U.S. (2003). The Role of Interfacial Layers in the Enhanced Thermal Conductivity of Nanofluids: A Renovated Maxwell Model. J. Nanopart. Res..

[46] Yang L., Xu X. (2017). A renovated hamilton–crosser model for the effective thermal conductivity of cnts nanofluids. Int. Commun. Heat Mass Transf..

[47] Patel H.E., Sundararajan T., Pradeep T., Dasgupta A., Dasgupta N., Das S.K. (2005). A micro convection model for thermal conductivity of nanofluid. Pramana.

[48] Mehrali M., Sadeghinezhad E., Latibari S.T., Kazi S.N., Mehrali M., Zubir M.N., Metselaar H.S. (2014). Investigation of thermal conductivity and rheological properties of nanofluids containing graphene nanoplatelets. Nanoscale Res. Lett..

